# The experiences of new graduate nurses and midwives going through a virtual interview recruitment process during the COVID-19 crisis: a cross-sectional study

**DOI:** 10.1186/s12960-021-00658-0

**Published:** 2021-10-02

**Authors:** Doreen Holm, Se Ok Ohr, Michelle Giles

**Affiliations:** 1 Nursing and Midwifery Services, Hunter New England Local Health District, PO Box 1743, Newcastle, NSW 2300 Australia; 2 Nursing and Midwifery Research Centre, Hunter New England Local Health District, James Fletcher Campus, 72 Watt Street, Gate Cottage, Newcastle, NSW 2300 Australia; 3 School of Nursing and Midwifery, Faculty of Health, University of Newcastle, Newcastle, Australia

**Keywords:** Virtual interviews, COVID crisis, New graduate nurse, New graduate midwife, Recruitment, Face-to-face interviews

## Abstract

**Background:**

The annual recruitment of new graduate nurses and midwives is key to recruiting large numbers of staff with the right attitude, skills and knowledge who are the best fit for the organisation. Virtual interviews were undertaken in 2020 due to the surge worldwide in the COVID-19 crisis. This study evaluates those virtual interviews and explores the sustainability of the model.

**Methods:**

A cross-sectional study was conducted at a large health organisation in New South Wales, Australia. Data were collected over 3 weeks using two online surveys, one for interviewees (*n* = 512) and the other for interviewers (*n* = 68). Quantitative data were analysed using descriptive statistics and frequency distributions, and additional free-text comments were analysed using content analysis.

**Results:**

Response rates were 55% (*n* = 280) interviewees and 54% (*n* = 37) for interviewers. The majority of interviewees (58%, *n* = 184) and interviewers (78%, *n* = 29) stated the interview was seamless or very seamless and 55% (*n* = 156) of interviewees and 73% (*n* = 27) of interviewers agreed interviewees conveyed themselves well during interviews. Over half of interviewees (65%, *n* = 182) and interviewers (51%, *n* = 18) agreed the virtual interview was fair or very fair for interviewee performance, regardless of age, race, or socio-economic status. However, many expressed a need for better internet access, equipment, and support, and a longer interview time to personally connect. Both new graduate interviewees (60%) and interviewers (75%) agreed virtual interviews are a suitable model for future use. However, some respondents indicated they preferred face-to-face interviews.

**Conclusions:**

The use of virtual interviews to select new graduates is considered acceptable, cost-effective and sustainable, as well as fair by the majority of participants. Study findings inform policy development, future planning, support the use of flexible selection practices and provide other health care professionals with a virtual recruitment model to consider when developing strategies to grow their future health workforce.

## Background

Annual recruitment of new graduate nurses and midwives (NG) is costly and time consuming for health organisations and the many NGs who enter the health workforce. This process has been highlighted as stressful and challenging, especially for those entering the workforce for the first time [1]. A vital component of the recruitment process is an individual interview which provides the opportunity to assess and determine if the potential recruit has the right attitude, skills and knowledge and will be a good fit for the organisation. In New South Wales, Australia the recruitment process for new graduate nurses and midwives to the facilities and services located across the state within Local Health Districts and Specialty Networks occurs annually, and is coordinated centrally by the NSW Health Nursing and Midwifery Office [2].

Since 2010, NG applicants applying for employment opportunities with the local health district (LHD), where this study was based, has progressively grown from 270 to a record number of NGs (*n* = 512) in 2020. The NGs applied for 233 positions available across 21 metropolitan, rural and remote health care settings within the LHD. Interviewing and processing of such large numbers is extensive and requires the allocation of a lot of time and resources by the LHD. Interviews have historically been held in predetermined locations across the LHD over several weeks. However, selection of appropriate candidates was to occur in August 2020 for employment into Grad Start Transition to Professional Practice positions commencing February the following year (2021) [2].

Traditionally, all NG interviews have been individual face-to-face interviews with the occasional phone interview for applicants who were not able to attend face to face [3–6]. In 2020, the COVID-19 pandemic was escalating within the LHD at the time the NG face-to-face interviews were planned, and there was an urgent need to rethink the way interviews would be conducted. The LHD needed to strengthen the health system through clinician recruitment, and at the same time deal with the increased pressure of providing additional services as the number of COVID-19 cases increased [7]. Releasing senior clinicians to conduct new graduate interviews was difficult, as they supported front line staff. However, the NG interviews were a critical part of the local Nursing and Midwifery workforce plan [8] so they could not be delayed. As a result in 2020, the virtual interview model was chosen for new graduate interviews.

The previous use of virtual interviews has predominantly been for the recruitment of medical officers, and those activities indicated that virtual interviews are an effective recruitment model [9–11]. There is also a suggestion that the use of virtual interviews is increasing because of continual technological advancements [4, 9, 12]. The advantages and disadvantages of virtual interviews have been discussed widely in recent literature. Some identified advantages are they place less financial and logistical burden on organisations [6, 13, 14] and reduce travel time and expenses for interviewees [15]. However, there have been some concerns highlighted which suggest virtual interviews restrict engagement and personal interaction opportunities [10, 16]. Further, the literature outlines the need for good technological equipment, a viable platform and an application such as Microsoft Skype^®^ for Business or ZOOM^®^ is needed to facilitate a successful virtual interview process [3–5, 13, 14]. In addition, the virtual interview process should ensure that it runs seamlessly so the interviewee is able to convey themselves effectively during the interview and that the process is fair for all interviewees regardless of their age, race, socio-economic status or location [9, 13].

Highlighted in the literature is an urgent need for rigorous evaluation of digital tools for recruitment and retention [4]. In general, recruitment and selection initiatives are rarely evaluated [17]. To the authors' knowledge, there was no available literature related to nursing and midwifery recruitment when a search using key words, virtual interviews or video conferencing interviews was undertaken.

During the height of the COVID-19 pandemic in 2020, a virtual interview model to recruit and select NGs was developed and implemented in the LHD where this study was based, similar to other studies [6, 11, 12]. Virtual interviews had never been performed on such a large scale in the LHD previously. A comprehensive plan was developed and implemented considering the insights gained from existing literature. A rigorous virtual interview process was developed and included strategies that considered fairness for interviewees, ease of access to and use of technology and adequate information and support [3, 4, 9, 13]. Little is known about how virtual interviews using Microsoft Skype^®^ for Business affect interviewees and interviewers in a large-scale recruitment process or the resources that are required to do so.

This paper presents findings from a cross-sectional evaluation study exploring the experiences and challenges of those who participated in the virtual interview process, from both the interviewers and interviewees perspectives. The future use of virtual interviews as a sustainable model for NG recruitment is also a pressing question given the persistence of the COVID-19 pandemic and advances in technology.

## Methods

This cross-sectional study aimed to explore the experiences of new graduate interviewees and interviewers, to identify the challenges and barriers of using the virtual model for selection interviews, and to assess the viability of that model for future use in NG selection.

### Setting

A LHD that provides health services to over one million people in acute and community health care settings in metropolitan, rural and remote locations across a large geographically dispersed area.

### Participants

Potential participants were identified from the NSW Ministry of Health new graduate application website for those applying to this LHD where the study was conducted, and the LHD was their first choice for employment. They were subsequently invited to participate in the online survey and the invitation was sent via their student / work emails, including an information letter describing the study and a link to the survey. Two groups were targeted in this study.

- 512 new graduate nurses (*n* = 473) and midwives (*n* = 39) applying for new graduate positions and who participated in virtual interviews during the week commencing the 10^th^ August 2020.
- 68 nurse and midwife interviewers participated in the new graduate virtual interviews. Interviewers consisted of senior nurses and midwife clinicians, educators and managers.

### Virtual interview

Due to the COVID restrictions at the time all new graduate interviews were performed virtually. Consistent with the existing literature [6, 10, 11], the following process for virtual interviews were planned and implemented:

#### Pre-interview:

• Provision of an information package and the virtual interview process.

• Interview process was reduced from 13 to 6 days.

• Pre-interview communication sent to universities and health staff, and opportunities for health staff training sessions and testing of equipment occurred.

• Interview scheduling reflected the applicants’ self-allocation to an interview panel, date, and time which occurred via an online appointment system. An email notification of the interview details included the individual interviewees and interviewers.

• A fact sheet was attached with instructions to facilitate ease with connecting to the interview via Microsoft^®^ Outlook^®^ calendar or alternatively through the NSW Health site.

#### During interviews

• Interview process—applicant identity was checked, and five interview questions were asked, scored, and the overall score ranked.

• Timekeepers managed the flow of the interviews by strictly adhering to time limits.

• Semi-structured debriefings for interviewers were held at the end of each interview day.

#### Post-interviews

• Recruitment coordinator and technology support team were available to support interviewees and interviewers.

### COVID-19-specific interventions

The majority of interviewers (*n* = 62) were located in one building except for a small number of interviewers (*n* = 6) which were located in rural facilities across the LHD. There were two interviewers per panel, and each panel was scheduled ten interviews per day. In addition, there was one panel that operated in the early part of the following week to accommodate any applicant overflow from the previous week, or any interview that needed rescheduling. Once the actual stability and capacity of the process was thoroughly tested, the number of interview panels increased daily, until finally 11 panels operated simultaneously each day. Each interview panel was allocated an interview room to ensure adequate social distancing, and the rooms were set up with computer technology and interview materials. Daily cleaning of rooms and equipment occurred each morning prior to commencement of interviews, as required by COVID-19 infection prevention protocols. The recruitment coordinator and the administration team audited all interview documentation, interview scores and referee checks were performed before positions were allocated. All documents related to the recruitment and interviews were entered into the LHD online recruitment and on-boarding system.

### Data collection

Data were collected via two different online surveys over three weeks during October–November 2020 (Additional file 1). The surveys were developed by modifying existing questionnaires based on the key constructs of seamlessness, fairness, and ability to convey themselves in the interview, as identified in an extensive literature review [3, 9, 10]. Further, other questions were developed to evaluate the virtual interview process. Both surveys included the same 16 questions identifying demographics, pre interview preparation, use of technology, the interview process; and potential for future use. These questions were structured as either multiple-choice questions or as a Likert scale where respondents were asked to choose an option from 1 (strongly disagree or not at all seamless) to 5 (strongly agree, very seamless). The respondents were provided with free-text fields on some questions so that they could make additional comments.

Both surveys were then developed in the SelectSurvey.NET software [18]. The online survey was then piloted on five nurse and midwife clinicians and educators to gain feedback on content clarity and relevance and modifications were made based on the feedback. The survey remained open for three weeks and a reminder email invitation was sent ten days after the initial email was circulated.

### Data analysis

Quantitative data are presented using frequencies and cross-tabulations. Responses to online surveys were downloaded from Select Survey^®^ into.csv files. Excel^®^ was used to summarise the data, preparing frequencies and cross-tabulations.

Free-text comments were analysed using content analysis by observation, which required reading and categorising comments one by one [19].

### Ethical considerations

Approval to conduct this study was granted by the LHD Human Ethics Committee (Reference No: 2020/ETH02470). Confidentiality was ensured as survey respondents were not identifiable in the online survey.

## Results

The response rates were 55% (*n* = 280) out of a possible 512 interviewees and 54% (*n* = 37) out of 68 interviewers. Of the interviewee respondents, 92% (*n* = 258) were Nursing applicants and 7% (*n* = 20) Midwifery applicants and a further 1% (*n* = 2) were dual degree applicants who were to graduate with both Nursing and Midwifery qualifications. The majority of interviewer respondents were senior nursing personnel who came from a diverse range of clinical and non-clinical roles; with 57% (*n* = 21) of interviewers working in Clinical Nurse Educator, Nurse Educator, Clinical Midwifery Educator and Midwifery Manager roles. The following characteristics of both respondent groups are detailed in Table 1 below.

**Table 1:** Characteristics of respondents

	Interviewees (*n* = 280)	Interviewers (*n* = 37)
	Percentage (number)	Percentage (number)
Age		
20–29	72% (202)	5% (2)
30–39	17% (48)	19% (7)
39–49	6% (18)	43% (16)
Over 50 years	4% (12)	32% (12)
Gender		
Female	89% (248)	81% (30)
Male	10% (29)	16% (6)
No answer	1% (3)	2% (1)
Aboriginality		
Yes	4% (11)	0% (0)
No	95% (265)	100% (37)
Prefer not to answer	1% (4)	0% (0)
Location of work		
Metropolitan area	56% (158)	84% (31)
Rural and regional area	44% (122)	16% (3)

## Usefulness of the infromation provided for the virtual interviews

The majority of interviewees (91%, *n* = 254) and interviewers (70%, *n* = 26) agreed that the information provided before the interview was useful in preparing for the interview. Most interviewee (90%, *n* = 252) and interviewer respondents (68%, *n* = 25) agreed or strongly agreed that the information provided to prepare for the use Microsoft Skype^®^ for Business was useful. The majority of interviewers (68%, *n* = 25) (strongly) agreed that redirection of new graduate program inquiries to the Interview Centre Coordinator was helpful, 24% (*n* = 9) remained neutral.

## Use of technology

To explore the ease of use of the virtual technology platform, five questions were asked: (1) technology (Microsoft Skype^®^ for Business) was easy to use; (2) the quality of the Microsoft Skype^®^ for Business was acceptable (voice and audio, visual clarity); (3) I was confident using Microsoft Skype^®^ for Business for the interview process; (4) the support provided during the interview was adequate, and (5) the connection to the internet network was stable. Overall, 79% (*n* = 222) of interviewees and 73% (*n* = 27) of the interviewers responded to those questions as ‘strongly agree’ or ‘agree’. However, 10% of interviewees and interviewer respondents stated that the use of the technology was an issue (see Table 2).

**Table 2:** Use of technology

	Interviewees					Interviewers				
	SA	A	N	D	SD	SA	A	N	D	SD
Technology Microsoft Skype^®^ for Business was easy to use	44% (122)	44% (123)	6% (18)	5% (13)	1% (4)	32% (12)	57% (21)	5% (3)	5% (3)	0% (3)
The quality of the Microsoft Skype^®^ for Business was acceptable	32% (87)	38% (105)	17% (48)	10% (27)	5% (13)	11% (4)	62% (23)	16% (3)	8% (3)	3% (3)
I was confident using Microsoft Skype^®^ for Business for the interview process	31% (88)	38% (106)	21% (58)	8% (19)	3% (9)	32% (12)	49% (18)	8% (3)	11% (4)	0% (3)
The support provided during the interview was adequate	48% (134)	39% (109)	9% (26)	1% (3)	3% (8)	41% (15)	51% (19)	8% (3)	0% (3)	0% (3)
The connection to the internet network was stable	43% (121)	36% (101)	8% (21)	10% (28)	3% (9)	24% (9)	49% (18)	14% (3)	11% (4)	3% (3)

SA: strongly agree, A: agree, N: neutral, D: disagree, SD: strongly disagree

## Seamless, being able to convey and fairness of virtual interviews

As suggested in the literature [9, 16], the study examined three aspects of the virtual interview: how seamless was the interview, how well interviewees conveyed themselves in the interview and fairness of virtual interviews for applicant performance.

**Fig. 1:**
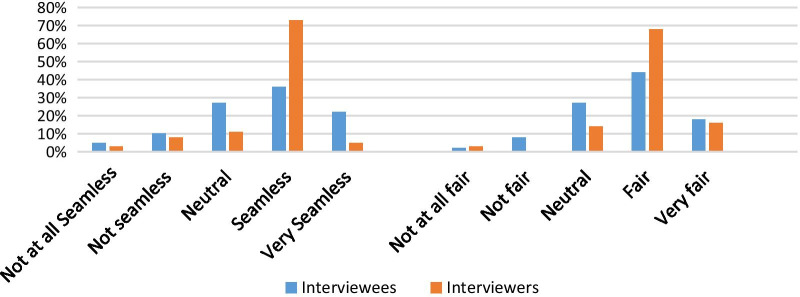
Seamlessness and fairness of virtual interviews

**Fig. 2:**
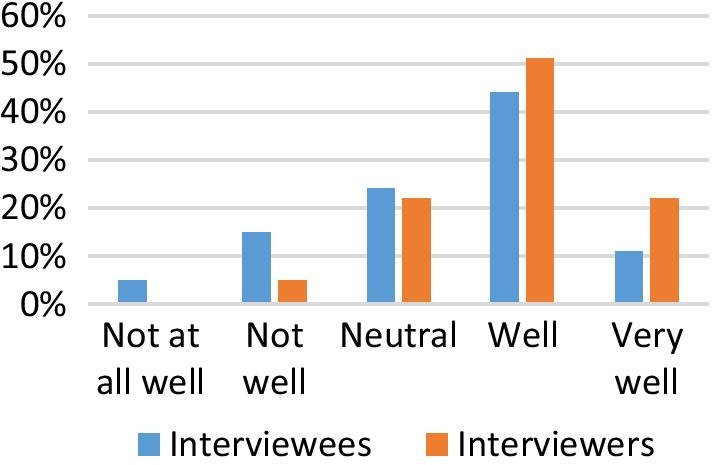
Able to convey themselves during interview

Many respondents (58% (*n* = 164) of the interviewees and 78% (*n* = 29) of interviewers) believed that the interview process was seamless or very seamless (Fig. 1). The majority of the interviewee and interviewer respondents agreed the virtual interview was fair or very fair for the performance of applicants in the interviews, 62% (*n* = 175) and 84% (*n* = 31), respectively (Fig. 1). A total of 73% (*n* = 27) of interviewer respondents observed that interviewees were able to perform and convey themselves effectively during interview, while 55% (*n* = 156) of interviewees agreed that they conveyed themselves well (Fig. 2).

Forty-one interviewees and 11 interviewers made additional text comments related to the fairness of the virtual interviews. Access to, and skills relevant to the use of technology were most frequently mentioned, with some respondents expressing concerns that more mature students and those socially disadvantaged may experience more difficulty with the virtual interview process.

*“Some people may have been put at a disadvantage, due to lack of access to a computer, internet services, or knowledge of how to operate the program. These issues could have increased stress for the applicant causing them to be more anxious and nervous during the interview.” Interviewee*

*“I believe for some it may be stressful as they do not have their own laptop or suitable device for interview. So socioeconomically this could place someone at a disadvantage but most universities, if informed of this, would ensure that the student was able to use a suitable space and device in order to attend their interview.” Interviewee*

## The future model for new graduate recruitment selection

**Fig. 3:**
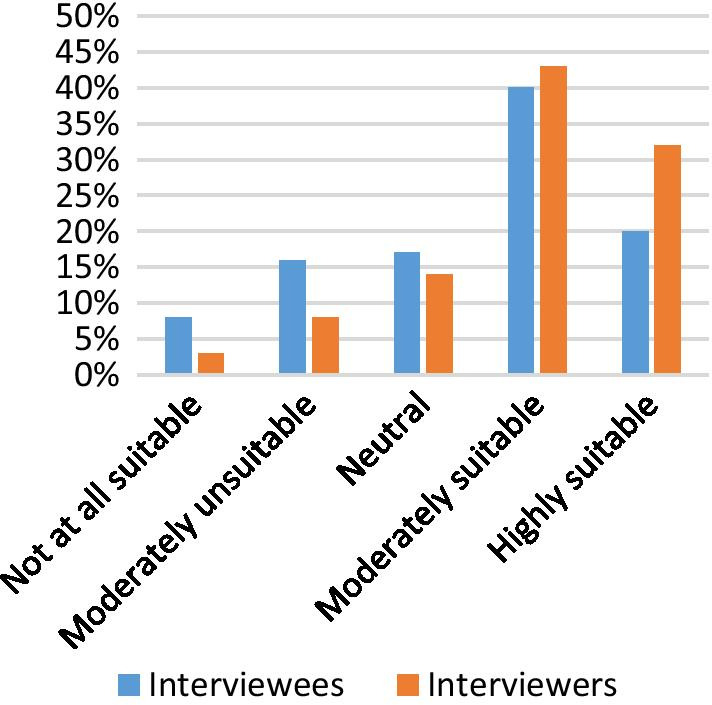
Sustainability for the future

When asked if ‘a virtual interview process would be a sustainable model for the future for NG interviews’, the majority of interviewees (60%, *n* = 166) and interviewers (75%, *n* = 28) stated that they believe virtual interviews are a sustainable future model (Fig. 3).

When asked to provide reasons for their response about the sustainability of the virtual interview model, there were different views expressed by 103 interviewees and 11 interviewers who made additional comments in the free-text fields. They were categorised under: face-to-face interview vs virtual interview; personal and impersonal, and the need to be connected and availability of workable technology. Fifty respondents preferred face-to-face interviews and the main reasons stated were that interviews need to be more personal and people needed to connect with one another at that level during the interview. In addition, the importance of appearance and body language were highlighted as essential elements when developing rapport. On the other hand, workable technology was mentioned in 26 comments as a “must’ for virtual interviews and 25 respondents stated convenience and flexibility as the reasons for preferring virtual interviews. Eighteen interviewees stated that being interviewed virtually made them feel less nervous and tired, as the virtual interview did not involve travel and was less stressful.

*“Virtual interviews are less hassle, as there is no stress about having to drive to the town or city for the interview. However, there are some limitations for people with limited technology understanding, and those who don't own a computer are impacted negatively.” Interviewee*

*“The virtual interview in the current times was necessary for the safety of everyone, however I do not feel I could convey myself and show who I am as a person” Interviewee*

In contrast, the majority of interviewers believed that students preferred the virtual interview as reflected in the following comment:

*“Students seemed calmer in themselves, less nervous and didn’t have any extra worry about getting to the interview centre, parking *etc*.” Interviewer*

### Respondent recommendations for improvement

Ninety (32%) interviewees and 16 (43%) interviewers made suggestions about improving the virtual interview process. They were categorised into the need for better technology, more interview time and personal support. The most mentioned improvements were related to the use of technology, in particular, consistent internet connection, better equipment and more information about the virtual interview process, so that they were more confident to use the technology.

*“Seamless internet, user interface, and technical development would be the next improvement factor for the interview.” Interviewee*

*“Perhaps more information about how to access the virtual interview. My personal issue was due to not having the correct Adobe* [Adobe^®^ Acrobat^®^ software] *required for the interview. The only other issue was that the interviewers were struggling to hear me at times, so perhaps a headset with a microphone would be beneficial to recommend to future interviewees.” Interviewee*

Although interviewees and interviewers agreed that virtual interviews are sustainable for use in the future, many interviewee respondents preferred having a different option ZOOM^®^ and FaceTime^®^ in addition to the platform used which was Microsoft Skype^®^ for Business.

Another improvement that both groups frequently suggested was more interview time. Some respondents stated they felt ‘very rushed’ and needed a longer period of time for the interview. The following quotes described the additional need for more time.

*“Needed a couple more minutes/breaks just to get the paperwork together and to take a moment, have a quick drink between interviews. I found this process worked my eyes and voice harder.” Interviewer*

*“Ensuring there is question time at the end as during my interview I did not get the chance to ask questions at the end of my interview.” Interviewee*

While interviewees explained that they found ‘the interviewers extremely supportive and helpful’ when there were technology issues during the interviews, some interviewees felt that the building of personal connections and rapport during the interview was limited.

*“Encourage interviewers to build a rapport. My interview process was very clinical and somewhat cold and uncomfortable which made me incredibly anxious.” Interviewee*

### Cost comparison between the traditional face-to-face interviews and virtual interviews

Historically, interviews were conducted at one interview centre in the metropolitan area and two separate interview centres in rural and regional areas. Travel to each interview venue in the geographically dispersed health LHD was a key part of the process, and incurred accommodation costs for the New Graduate Coordinator and Administrative support person/s ($2,000), with a health service vehicle being used for travel. Interviewees were expected to also travel to the designated interview centres and many incurred costs in doing so. The interviews traditionally ran over 13 days and given the large geographical area, an additional 2 days for travel was required.

Regional venue hire was cost neutral as the external provider collaborated with the LHD and the metropolitan and rural venues remain under LHD management. Catering expenses were covered by the LHD at a cost of approximately $1500 dollars and the staff (time) salary invested in the process was calculated at $75,788.54 dollars for a 13-day interview period for the historical face-to-face interviews.

With COVID-19 travel restrictions in place and the change to virtual interviews, there were no travel expenses incurred. The staff (time) and related salary for the New Graduate Interview Coordinator, administration staff [3] nursing and midwifery interviewers (68) was reduced to $32,046.34 for a six day interview period, although catering costs increased to $1800.00 due to an increase in the number of panels and subsequent interviewers.

## Discussion

This study explored the experiences of new graduate interviewees and interviewers who participated in a virtual interview as part of the annual new graduate recruitment process. Study findings provide valuable insights into the suitability of the virtual interview process in the annual NG recruitment and selection process, and the associated challenges and the possibility of sustaining this model into the future.

In the context of COVID-19 crisis, the virtual interview model was mainly driven by the consequences arising from the pandemic as described by Patel et al. [6]. The potential for harm minimisation and compliance with social distancing and public health requirements were reasons to choose and implement the virtual interview model for the selection of NGs. Important aspects in carrying out virtual interviews, as outlined by Deitte et al. [5] and Vining et al. [9], were adhered to, in that the virtual interview process was highly organised and modified from the normal face-to-face interview so that it was robust and consistent for all participants.

At the LHD level, to ensure adequate preparation and understanding of the change in interview process, modifications were made to enhance open communication, ensure all parties were familiar with the process and what was expected of them. The scheduling of interviews via Microsoft Skype^®^ for Business reflected the self-allocation of interview appointments made by interviewees. This provided consistency and devolved management of their time back to the interviewees, resulting in a smooth transition and less changes to interview schedules [5]. In addition, time keeping was a critical aspect and timekeepers managed the flow of the interviews by strictly adhering to time allocations which minimised scheduling conflicts [20].

The success of the virtual interview model is highly dependent on participants having stable internet access [10–12]. This is strongly reflected in the study findings for both interviewers and interviewees. Comments provided by both groups indicated it is essential that the equipment and internet connection are fit for purpose, work well and are stable. Similar to findings in other studies [4, 6], some respondents in this study perceived that there can be more challenges related to infrastructure and connectivity during the interviews in the geographically disperse study setting, and in particular, in rural environments. The quality of the interview experience relies on stable technology, user ability and the efficient use of computer software for both interviewees and interviewers. In light of this, adequate education within health organisations in the use of computer technology is a fundamental requirement for the creation of what Deitte et al. [5] described as a “robust digital environment”. The need for virtual readiness by lessening dependence on the capability of the users or the stability of the technology may provide a better quality interview experience [6].

This study conveys a mostly positive perception from both interviewees and interviewers, which is consistent with the findings from Vining et al. [9] and Saiegh et al. [16] where the recruitment of medical officers using virtual interviews was successful. The interviewees and interviewers in this study reported different perspectives related to virtual interviews being seamless and fair with interviewees rating them as being less seamless or fair. The qualitative comments expressed concern that older or socioeconomically challenged interviewees might face the potential for disadvantage depending on the availability of computer equipment and their lack of access or technological skills. However, the majority of interviewees in this study belonged to a younger demographic, and this age group is generally described as more technologically savvy, and accustomed to navigating virtually [21].

Some suggestions for future improvement in the virtual interview model were related to the time allocation and a need for more interview time. Interviewees said that they had no time to ask questions at the end of their interviews, and interviewers believe a short break between interviews would have been beneficial. In addition, extra time may have assisted in sorting out technological challenges and given more time to build greater rapport between the interviewees and interviewers. Allocation of additional time would enhance virtual interview attributes such as interviewer warmth, and question standardisation as described by Kohn and Dipboye [22] and be conducive to applicants being able to present and convey themselves better during the structured interview [9]. These recommendations will be considered in future recruitment and selection of nurses and midwives, in conjunction with the recommendations of the existing literature to maximise the virtual interview experience [4, 11, 12].

In line with several studies that have identified the cost effectiveness of virtual interviews [9, 23, 24], the findings of this study established that the LHD virtual recruitment model expenses were half that of the face-to-face interviews. Therefore, from the LHD’s perspective, the model may be a cost-effective alternative for future selection to NG positions.

The findings of this study clearly indicate that the virtual interview model was an effective model for the extensive annual NG recruitment and selection process during the COVID-19 pandemic. In addition, given the added advantages of convenience, less travel and less resources required, this virtual model is viable and should be considered for future large-scale recruitment regardless of pandemic restrictions. The development and implementation of the virtual interview process has been the stimulus to thoroughly review and rethink how NG recruitment and selection interviews are conducted within the LHD. The future of NG virtual interviews as a stand-alone model or as part of a mixed-method approach combined with face-to-face interviews will require further study to build on best practice.

## Limitations and strength of the study

Although this study was based in only one LHD in NSW Australia, the findings can reasonably be generalised to other NG recruitment sites across the state and be considered as a viable and effective way to recruit other new graduate professional groups in health. The utility of "virtual interviews" may also be limited in jurisdictions or countries with less advanced technology or access to virtual technology infrastructure or power systems.

## Conclusions

The use of the virtual interview recruitment and selection model for NGs has been identified as a viable alternative to the traditional face-to-face interview process. Virtual interviews are perceived to be fair and acceptable to users and are shown to utilise only half the resources of the traditional recruitment processes. In times of resource scarcity this is a significant advantage. Study findings can inform decision-makers on policy development, future planning and influence the implementation of evolving recruitment and selection practices. At the same time findings from this study may be a catalyst to ensure there is adequate education about virtual platform use and mandates the need for a stable technological environment to conduct interviews.

## Data Availability

The datasets generated and/or analysed during the current study are not publicly available due to privacy issues, but are available from the corresponding author on reasonable request.
